# Effectiveness guidance document (EGD) for acupuncture research - a consensus document for conducting trials

**DOI:** 10.1186/1472-6882-12-148

**Published:** 2012-09-06

**Authors:** Claudia M Witt, Mikel Aickin, Trini Baca, Dan Cherkin, Mary N Haan, Richard Hammerschlag, Jason Jishun Hao, George A Kaplan, Lixing Lao, Terri McKay, Beverly Pierce, David Riley, Cheryl Ritenbaugh, Kevin Thorpe, Sean Tunis, Jed Weissberg, Brian M Berman

**Affiliations:** 1 Institute for Social Medicine, Epidemiology and Health Economics, Charité University Medical Center, Berlin, Germany; 2 Center for Integrative Medicine, University of Maryland School of Medicine, Baltimore, Maryland, USA; 3 Department of Family and Community Medicine, University of Arizona, Tucson, USA; 4 Patient stakeholder; 5 Group Health Center for Health Studies, Seattle, USA; 6 Department of Epidemiology and Biostatistics, University of California, San Francisco, USA; 7 Research Department, Oregon College of Oriental Medicine, Portland, Oregon, USA; 8 International Academy of Scalp Acupuncture, Southwest Acupuncture College, Santa Fe, USA; 9 Center for Social Epidemiology and Population Health, University of Michigan, Ann Arbor, USA; 10 The Institute for Integrative Health, Community programs, Baltimore, USA; 11 Integrative Medicine, University of New Mexico Medical School, Albuquerque, USA; 12 Dalla Lana School of Public Health, University of Toronto, Toronto, Canada; 13 Center for Medical Technology Policy, Baltimore, Maryland, USA; 14 Kaiser Foundation Health Plan and Hospitals, Hospitals, Quality and Care Delivery Excellence, Oakland, USA

**Keywords:** Comparative effectiveness research, Effectiveness guidance document, Acupuncture

## Abstract

**Background:**

There is a need for more Comparative Effectiveness Research (CER) to strengthen the evidence base for clinical and policy decision-making. Effectiveness Guidance Documents (EGD) are targeted to clinical researchers. The aim of this EGD is to provide specific recommendations for the design of prospective acupuncture studies to support optimal use of resources for generating evidence that will inform stakeholder decision-making.

**Methods:**

Document development based on multiple systematic consensus procedures (written Delphi rounds, interactive consensus workshop, international expert review). To balance aspects of internal and external validity, multiple stakeholders including patients, clinicians and payers were involved.

**Results:**

Recommendations focused mainly on randomized studies and were developed for the following areas: overall research strategy, treatment protocol, expertise and setting, outcomes, study design and statistical analyses, economic evaluation, and publication.

**Conclusion:**

The present EGD, based on an international consensus developed with multiple stakeholder involvement, provides the first systematic methodological guidance for future CER on acupuncture.

## Background

Effectiveness guidance documents (EGDs) provide specific recommendations about the design of prospective studies that will inform decisions by patients, clinicians and payers [1]. The recommendations are targeted to clinical researchers conducting studies of specific types of interventions or clinical conditions. EGDs are intended to be analogous to Food and Drug Administration (FDA) guidance documents in the US, but are focused on design elements intended to support clinical and health policy decision-making. The recommended methods aim to achieve a balance between internal validity, relevance and feasibility. These documents are developed through an extensive consultative process involving a broad range of experts and stakeholders.

Comparative Effectiveness Research (CER) is the generation and synthesis of evidence that compares the benefits and harms of different treatment options to prevent, diagnose, treat, and monitor a clinical condition or to improve the delivery of care. The purpose of CER is to assist consumers, clinicians, purchasers, and policy makers to make informed decisions that will improve health care at both the individual and population levels [2]. In addition, the Institute of Medicine (IOM) used six characteristics to describe CER [3]: 1) Informing Informs a specific clinical decision from the patient perspective or a health policy decision from the population perspective, 2) compares at least two alternative interventions, each with the potential to be “best practice”, 3) describing results at the population and subgroup levels, 4) measures outcomes—both benefits and harms—that are important to patients, 5) employs methods and data sources appropriate for the decision of interest, 6) is conducted in settings that are similar to those in which the intervention will be used in practice.

There is a strong need for additional CER for disorders that are common and costly to society and that have a great degree of variation in their treatment. [4] Acupuncture is one of the complementary and alternative therapies used for patients suffering from chronic symptoms, such as pain [5]. Guidelines for designing and reporting CER involving acupuncture are needed since acupuncture treatment varies among different types of providers, states and countries [6], and the design and results of previous studies have been highly variable.

“Effectiveness” is a measure of the extent to which an intervention, when deployed in the field in routine circumstances, does what it is intended to do for a specific population [7] and therefore can be of high relevance to policy evaluation and the health care decisions of providers and patients. In contrast, “efficacy” refers to the extent to which a specific intervention is beneficial under ideal conditions [7]. It is important to note that there is often no sharp distinction between efficacy and effectiveness trials [6]. Rather, different dimensions of trial design may be distributed at varying sites across the continuum. For randomized trials, the distinction between explanatory and practical or pragmatic is also often made [8-10]. However, within this document we will use the more general terms “efficacy” and “effectiveness” to label both ends of the continuum and a trial that is more on the effectiveness side would be a pragmatic trial.

Traditional medical systems such as Chinese medicine frequently use multiple interventions, many of which are individually complex, e.g. an herbal formula or an acupuncture point combination. Such complex interventions, whose components may act additively or synergistically, are difficult to evaluate. Yet, the aspect of CER that addresses effectiveness in a more everyday practice setting suggests that studies can be designed to compare real-world whole systems of health care. The Medical Research Council guidance in the UK provides the following advice for developing and evaluating complex interventions [11]:

A good theoretical understanding is necessary to understand how the intervention causes change, so that weak links in the causal chain can be identified and strengthened.

Lack of effect may reflect implementation failure rather than genuine ineffectiveness; a thorough process evaluation is needed to identify implementation problems.

Variability in individual level outcomes may reflect higher level processes; sample sizes may need to be larger to take account of the extra variability. Cluster randomized designs may also be considered.

A single primary outcome may not make best use of the data; a range of measures will be needed and unintended consequences accounted for where possible.

Ensuring strict standardization may be inappropriate; if a specified degree of adaptation to local settings is allowed for in the protocol, the intervention may work better.

The aim of the present EGD is to provide comprehensive, consensus-based guidance for future acupuncture studies by taking existing guidelines into account. This document should be seen as part of a larger research framework that would include translational research strategies to enhance the likelihood that mechanistic studies, clinical studies and clinical practice will each inform and be informed by one another.

## Methods

The development of the EGD followed a clear predefined process and included an initial written Delphi round on the methodological remarks and overall structure of the document, followed by a one-day consensus development workshop (July 24, 2011 Santa Fe, USA) and two written post-workshop Delphi rounds to finalize the document. Participants of the workshop had following background (2 acupuncture patients, 1 health insurance representative, 4 medical doctors (one of them providing acupuncture), 2 acupuncture practitioners with Asian background living in the US, 1 nurse, 7 methodologist with background in statistics or epidemiology or neuroscience). Half of them had long-term experience in acupuncture research. In the consensus meeting an adapted the “World Café” methodology was used. The World Café method as developed by Brown and Isaac is a simple, effective, and flexible format for hosting large group dialogue [12]. It allows a collaborative dialogue, sharing knowledge, community participation and suitable for a setting that involves different stakeholder groups. Participants are asked to sit in one of several table rounds and discuss 20 to 45 minutes about one or more predefined questions. At the end of each round, one person remains at each table as host, while the other participants move to other tables and form new groups. Table hosts welcome the newcomers, summarize the results of the table ’s conversation so far and ask new questions or go deeper into the original one. After three or more rounds, the whole group gathers in an auditorium and the table hosts present the results, all participants explore and discuss emerging themes and insights, which are captured on flipcharts or other means. The technique was used in our project in a more consensus-oriented way by sequentially narrowing the content of each question to a final decision. The first group was instructed to brainstorm on the topic, and come up with principles to answer the question. The second group debated and refined the principles, and suggested a few options. The third group made the final decision based on the deliberations of previous groups. Each table had a moderator who communicated a concise summary of each group to the next.

In addition, an international review by acupuncture research experts was employed at several stages of the EGD development process. After the pre-workshop Delphi round (before the consensus meeting) and after the two written post-workshop Delphi rounds the document was sent to an external international review board including eight experts from four countries (US, Germany, UK, Italy), who did not participate in the consensus development workshop.

The consensus process was finalized after all workshop participants and the external review experts consented.

To allow a comprehensive summary of the recommendations as well as to provide relevant methodological background information, the results of the consensus were structured into two parts 1) methodological remarks and 2) recommendations.

This EGD refers also to the following documents:

CONSORT for parallel group randomized trials [13].

CONSORT extension for pragmatic trials [8].

CONSORT for non-pharmacological trials [14].

CONSORT extension for cluster randomized trials [15].

STRICTA as a CONSORT extension for acupuncture studies [16].

SAR Board of Directors White Paper [17].

## Results

### Methodological remarks

This EGD for acupuncture is the first such document in the field of Complementary, Alternative and Integrative Medicine (CAIM) and may serve as a model for other CAIM interventions. Here we will describe the methodological issues that impact CER in acupuncture research.

#### Acupuncture as a complex intervention

Acupuncture is a component of Chinese medicine, which comprises a “whole medical system” using unique diagnostic and therapeutic approaches. [6] To date, only a few studies have evaluated the effectiveness of acupuncture as part of a complex Chinese medicine intervention [18]. In addition, acupuncture needling itself could be seen as just one component of a complex intervention. For research purposes, in their White Paper [17], the Society of Acupuncture Research Board of Directors divided the components of acupuncture treatments into the following groups:

Needling components (i.e., needle size, retaining time, depth, stimulation, location, frequeny)

Specific (acupuncture theory-related) non-needling components that are traditionally considered to have therapeutic value, for example, in Chinese Medicine the physical components such as palpation.

Generic, nonspecific non-needling components that are not unique to acupuncture treatments, such as belief and expectations of the practitioner and patient, therapeutic setting, time and attention.

#### Research strategy

For the field of complementary medicine, several authors have highlighted the importance of comparing different treatment options that reflect usual care [19,20]. Furthermore a research strategy has been suggested aimed at establishing comparative effectiveness before assessing component efficacy [20,21]. Using the proposed strategy will generate evidence relevant to clinical practice. It will also emphasize the important but sometimes subtle differences between CAM and conventional medical practice [21]. From a translational approach, it is recommended that acupuncture should be studied “top down” in real-world, multi-component “whole-system” trials, as well as “bottom up” in mechanistic studies that focus on causal pathways and on understanding how individual treatment components work and interact [17]. In this manner, clinical and basic research can inform one another, thereby benefiting clinical relevance and design of future research.

In order to provide a realistic assessment of treatment options in a usual care context, CER trials often include multiple comparison groups, more heterogeneous patients and longer-term outcomes. CER trials will also require additional time to engage stakeholders in study planning and to engage and train participating practitioners working in usual care. Cluster-randomized trials on acupuncture were recommended as one of the priorities for CER by the IOM [2]. and might help overcome recruitment problems. Pilot studies are an important instrument when planning CER to determine feasibility, test outcome measures and provide information for sample size calculation.

A Bayesian (adaptive) statistical approach might be an interesting option for real-world CER where it is “noisier” than in “efficacy” studies. Standard statistical (frequentist) techniques to determining study size require increased sample sizes in effectiveness studies, because of greater variability and the fact that when comparing several active treatments relatively small differences are expected [22]. Designs that use features that change or “adapt” in response to information generated during the trial can be more efficient by using formal, probabilistic statements of uncertainty based on the combination of all sources of information both from within and outside a study [22]. For further information see [23].

#### Research question and the efficacy-effectiveness continuum

When planning a study, it is important to determine the location on the efficacy-effectiveness continuum. In other words, should the study be designed primarily to exclude bias and increase the chance of detecting a specific effect or to reflect usual care?

Efficacy studies aim to produce results with high internal validity by reducing both bias (e.g. using blinding) and variation (e.g. narrow eligibility criteria and a standardized treatment protocol) to increase the likelihood of detecting the hypothesized difference. These studies aim to assess a treatment outcome under “ideal” conditions with highly selected patients. At the other end of the continuum, results from effectiveness studies aim to inform decision makers about the potential benefits of an intervention in a usual care setting, making the results of the study generalizable and relevant to routine care by studying patients within the context (methods and practitioners) in which the acupuncture treatment is usually deployed.

The place along the continuum is multi-dimensional and is influenced by the research question [24,25]. Since there is a range of relevant clinical research questions on acupuncture, each study context, outcome and design (e.g. nature of the comparison group) should clearly reflect the study question.

### Possible research questions are

#### Specific effect

Does acupuncture needling have acupuncture point specificity (i.e. is needling at real acupuncture points superior to a penetrating sham procedure at non-acupuncture points)?

Does acupuncture needling have a specific effect (i.e. is needling at real acupuncture points superior to a non-penetrating sham procedure at acupuncture points or non-acupuncture points)?

Does depth of needling, type of stimulation or other needling parameters have an effect on the outcome?

#### Acupuncture effect

Is acupuncture treatment superior or non-inferior to conventional standard treatment?

Is acupuncture treatment in addition to usual care or standard care superior to usual care or standard care alone?

What dose (number of treatments) of acupuncture is needed to see an effect?

#### Safety and costs

What are the types and frequency of acupuncture side effects?

Is acupuncture treatment cost-effective compared to another intervention?

Studies that aim only to determine the specific effect of acupuncture are not part of CER, because sham acupuncture is not considered an “active” comparison group or as a real treatment option. Only trials that compare a minimum of two treatment options are considered to represent CER [3]. However, a study that includes a standard care or usual care comparison group in addition to a sham acupuncture group can contribute to CER. There is an ongoing discussion about the challenges of using sham controls in acupuncture studies [17,26], which should be considered when planning those studies. When comparison groups labeled as “usual care”, “standard care,” or “best practice” are used, specific details of the interventions should be described.

A helpful tool for planning randomized trials is the PRECIS (pragmatic–explanatory continuum indicator summary) [10]), which includes the following ten dimensions that have an influence on the efficacy-effectiveness continuum: eligibility criteria of participants, flexibility of the intervention protocol, flexibility of the comparison group treatment protocol, expertise of the practitioners in the intervention group, expertise of the practitioners in the comparison group, adherence of practitioners, compliance of participants, follow-up intensity, outcome parameters, and analysis of study population.

Starting from these PRECIS dimensions [10] as well as the six characteristics of CER developed by the IOM [3], we propose to structure this guidance document a simplified list of five criteria that are most relevant for the study design of CER: participant selection/eligibility criteria, treatment protocols, practitioner expertise, outcomes, and setting in which the study is conducted.

The following paragraphs will introduce the methodological background for some designs aspects that are highly relevant for CER on acupuncture.

### Developing the acupuncture and comparison group interventions

The treatment protocols for the intervention and the comparison group are central aspects of the study. The STRICTA guidelines for reporting acupuncture trials should be taken into account when planning intervention and comparison group treatment protocols [16].

#### Acupuncture treatment protocol

The acupuncture intervention in CER could be a new intervention (e.g. new acupuncture style or microsystem) or an intervention that is already available (e.g. Chinese style needle acupuncture). When evaluating a new intervention, its characteristics (e.g. dosage, frequency, setting) should reflect the context in which it will be deployed in the future in a usual care context. When evaluating acupuncture as an existing intervention that is widely available for the external validity of the results, it is important that the treatment protocol of the acupuncture group reflects common practice to the extent possible. However, this could be difficult because of the large heterogeneity in acupuncture styles and expertise of the practitioners. Registries (see below) or prospective observational studies are helpful sources to inform researchers about practice in usual care. If this information is not available, cross-sectional surveys or consensus procedures with acupuncture experts are the next best option. However, when selecting the experts participating in the consensus, their expertise should reflect the heterogeneity of the acupuncture practised in usual care. If consensus is lacking before large randomized comparative effectiveness studies are performed, pilot studies comparing different treatments should be considered [27]. Intervention planning, should also take into account that acupuncture styles, as well as training and experience of practitioners can vary considerable in different states and countries.

#### Comparison group treatment protocol

The comparison group treatment protocol should be planned and developed with the same rigor and attention to detail as the acupuncture intervention. When standard treatment or usual care is used as the comparison group, the treatment components should be described in the study protocol and detailed usage during the study should be documented and reported.

#### Co-interventions

In CER, co-interventions (e.g. pain medications in pain trials) are often more heterogeneous than in efficacy trials, and for understanding the context of the study, it is relevant to document and report them.

### Registries

The Agency for Health Care Research and Quality (AHRQ) in the US has defined patient registries as an organized system that uses observational study methods to collect uniform data (clinical and other) to evaluate specified outcomes for a population defined by a particular disease, condition, or exposure, and that serves a predetermined scientific, clinical, or policy purpose(s) [28]. Registries are seen as a valuable complement to randomized controlled trials in determining real-world outcomes in the practice of medicine and do not generally have restrictive inclusion or exclusion criteria, nor do they specify the therapy to which the health care provider must adhere. They can be used to evaluate outcomes for diverse purposes ranging from the natural history of a disease, to the safety of drugs or devices, to the real-world effectiveness of therapies. These patient registries are designed to answer predefined questions by choosing a study design, measurable outcomes, the study population and analysis. In the registry design, potential sources of bias (systematic error) should be addressed to the extent that is practical and achievable [28]. Population-based registries are the most desirable kind of registry, because they minimize selection of participants.

Another approach is to use existing data, usually from the electronic medical records of a well-defined health care delivery system, for comparative effectiveness research [29]. This line of research tends to use statistical methods that differ from the clinical trial- based approaches in AHRQ-type registries, including both propensity matching for synthetic trial designs and Heckman’s selection models [30], but these methods are more difficult to employ than conventional analyses and methodological problems remain.

An important reason for extending acupuncture CER research into these domains is the opportunity to evaluate this complex intervention as it is actually practiced, in the types of patients who are willing to use it, and in the settings where it is generally used.

### Allocation methods

Although CER promotes registries and observational studies in addition to randomized trials, randomized studies are still considered the most robust method for generating comparative effectiveness evidence, as they are more likely to control for bias and confounding. The randomized trial will undoubtedly remain an important component of an advanced CER framework [22]. It is essential that the treatment groups are comparable before treatment. Randomization as a stochastic method has historically been the method of choice and is still the most frequently used method. To prevent selection bias it is important to conceal the allocation sequence from those assigning participants to intervention groups, until the moment of assignment. This prevents researchers from (unconsciously or otherwise) influencing which participants are assigned to a given intervention group [31].

Dynamic allocation procedures could be an alternative to randomization procedures. Depending on the study design, they could balance treatment arms across baseline prognostic factors for clinical trials more effectively than randomization [32,33]. Dynamic allocation uses algorithms to determine each subsequent patient’s assignment in a manner that produces the best overall balance between treatment groups. Design-adaptive allocation methods (minimization) use only patients’ baseline characteristics to determine the allocation of the next patient [34]. Assignments using minimization are more predictable than with randomization, but there are ways to avoid this (see Taves for rank minimization [35]). Minimization is gaining more acceptance and was recognized by CONSORT as an acceptable alternative to random assignment [36].

Response-adaptive allocation algorithms use both previous patients’ baseline characteristics and outcomes. There is a lack of successful studies, especially in acupuncture research, using these response-adaptive allocation approaches.

Although methodologically important, allocation methods create an artificial context that may influence the outcome, e.g. they could have a direct impact on the selection of participants. Treatment preferences that are influenced by patient expectations are considered a relevant determinant of the placebo effect [37]. For example, if patients’ preferences have a significant influence on whether they choose to participate in a study and how they respond to treatment, the findings of randomized trials may not apply to those patients who would avoid participating in trials and instead seek other options in usual care. Several designs have been developed to investigate whether preferences matter. The most important is probably the partially randomized patient preferences design. In such trials, eligible patients are asked whether they have a preference for one of the two (or more) treatments and whether they agree to be randomized. Those not giving consent to randomization receive the treatment of their choice, while those without strong preference are randomized [38]. On an exploratory level this design provides information about whether the results observed among randomized patients are different from those among patients who were not randomized because of strong treatment preferences. A number of large acupuncture trials have used variants of this design, including incentives to motivate patients to accept randomization [39-43].

Randomization methods including both simple and stratified randomization could be used to allocate patients to the treatment groups. Stratified randomization is a useful approach to control for relevant factors for imbalances in treatment groups. In settings where randomization at the patient level is not feasible, cluster randomization might be an option. Cluster trials randomize social units, such as households, primary care practices, hospital wards, classrooms, neighborhoods and entire communities, to different intervention arms. Compared with randomized trials at a patient level, cluster trials have less statistical power while using the same number of patients [44]. Because cluster randomization is often applied to an entire hospital or clinic population without exclusion, it enhances recruitment and generalizability. Cluster trials allow resources to be used to best advantage and due to this they are likely to be less expensive than the “traditional” randomized controlled trial. However, cluster-randomized trials also have limitations, for example, selection bias can occur and withdrawal drop-out of whole clusters may seriously affect the validity of the results. For further guidance on cluster-randomized trials see [44].

### Blinding and expectation

Blinding reduces bias by minimizing the possibility that the results can be affected by expectations of patients and practitioners. It is commonly accepted that practitioners cannot be blinded to the acupuncture intervention. In contrast, the intent to blind patients is common practice in efficacy trials that compare acupuncture with sham acupuncture, even though data from sham-controlled acupuncture trials suggest that patients’ expectations might influence the treatment outcome [45]. Further, patient blinding seems to be impossible when comparing acupuncture with usual care or standard care. Thus, in this latter type of CER trial, where neither practitioner nor patient is blinded and the outcomes are not assessed by the patient, it is important to include an assessor for the relevant outcome who is blinded to the patients’ group assignment. This is not possible in trials that use patient self-rated outcomes such as chronic pain measures or quality of life. In such cases 1) questionnaires sent to the patients’ home or 2) or blinded telephone interviewers could help to assure that the practitioners do not influence patients’ outcome assessments. Another option is to assess patients’ and practitioners’ expectations and to use them as covariates when analyzing the data to adjust for different expectations in the treatment groups.

### Health economic evaluations

Over the last few years, health economic data has become increasingly relevant for decision makers faced with the challenge of reconciling the growing demand for healthcare services with the funds available [46]. The number of economic evaluations of complementary and integrative medicine has increased over the last few years and the largest number has been done for acupuncture either in addition or compared to routine care [47]. There are a variety of best practices for performing economic analyses that should be considered when incorporating costs into CER [48].

#### Recommendations for acupuncture comparative effectiveness research

These recommendations aim to support optimal use of resources for generating evidence that will inform stakeholder decision-making as fully as possible. They are based on the assumption that it is a conscious decision of the relevant stakeholders to reduce internal validity in order to increase generalizability, relevance, feasibility and timeliness of research results. Multiple stakeholders were involved in the consensus process for this EGD to balance aspects of internal and external validity in the recommendations.

#### Recommendations developed for the following fields are summarized in (see also Table 1)

**Table 1:** Checklist for CER relevant aspects for acupuncture clinical studies

**Designing CER studies**	
Stakeholder involvement	□ All relevant stakeholders are involved in
	□ Identification of research topic
	□ Plan and design of CER
	□ Interpretation of results
Efficacy-effectiveness continuum	□ Study location on the efficacy-effectiveness continuum is determined for participant selection/eligibility criteria, treatment protocol, practitioner expertise, outcomes, setting in which the study is conducted.
**Study population**	
Eligibility criteria	□ Study population reflects those who usually receive this treatment
	□ Study population includes both acupuncture- naïve and acupuncture-non-naïve patients
Patient recruitment	□ Patients are recruited from site where the treatment is usually employed
**Treatment protocol, expertise, and setting**	
Acupuncture intervention	□ Adequate consensus procedure is used to define intervention
Comparison groups	□ Adequate consensus procedure used to define comparison(s)
	□ Definitions and details for terms such as “usual care” provided
Treatment documentation	□ Documentation reflects which treatments were received in all groups (interventions and co-interventions)
**Outcomes**	
Measures	□ Widely accepted or standardized outcome measure used
	□ Secondary outcomes capture relevant patient-centered dimensions for the condition under study
Timing	□ Assessment schedule is balanced gaining study relevant data without substantially disrupting treatment protocol or setting
**Study design and statistical analysis**	
Allocation	□ Allocation is concealed
	□ Stratification for subgroups or dynamic allocation for key characteristics used
Blinding	□ Outcome data inaccessible to practitioners
	□ Blinded outcome rater, if possible, used
Preferences/ expectation	□ Preferences and expectations measured at baseline
Sample size	□ Sample size takes patient heterogeneity into account
	□ Required sample size is feasible
	□ Study has enough power for planned subgroup analyses
Subgroups	□ Relevant subgroups preplanned (e.g. gender)
	□ Exploratory subgroup analysis mentioned in study aims
Statistical analysis	□ Intention-to-treat analyses planned
	□ Relevant subgroup analyses planned
	□ Adjusted for stratification variables, baseline differences and relevant confounders
	□ Setting reflects reality in clinical practice
Methodological approach	□ Standard methods for economic evaluations used
	□ Sensitivity analysis for all relevant stakeholder perspectives
	□ Relevant subgroups identified
Observation time	□ In chronic diseases long-term observation (≥ 12 months), if possible, planned
Guidelines	□ Relevant guidelines (e.g. CONSORT) consulted and followed
Content	□ Stated how and why this is CER
	□ Study setting (incl. procedure of practitioner selection) described in detail
	□ Treatment group description from informed consent provided
	□ Comparison groups described in detail
	□ Data provided on all interventions and co-interventions received
	□ Relevant subgroup analyses reported

Designing CER studies

Treatment protocol, expertise and setting

Outcomes

Study design and statistical analyses

Economic evaluation

Publication

### Designing CER studies

1) Stakeholder engagement

a) Involvement of all relevant stakeholders (e.g. practitioners, clinicians, patients, payers, researchers) is highly relevant for CER.

b) Identifying relevant topics, planning and design, and interpreting study results should involve all relevant stakeholders.

2) Study question and the efficacy-effectiveness continuum

a) The study question should be clearly phrased, and include all relevant information about study participants, interventions, comparison groups and outcome parameters. In particular, it should clarify whether the acupuncture treatment is to be assessed as an “alternative” in direct comparison, for superiority or non-inferiority, to or as an adjunctive to a usual or standard care treatment.

b) During the trial planning phase, time should be given to discuss and determine the trial’s position in the efficacy-effectiveness continuum for the following aspects: participant selection/eligibility criteria, treatment protocol, practitioner expertise, outcomes, study setting. Using the more detailed PRECIS tool to support this process is recommended [10].

### Study population

3) Eligibility criteria

a) In the context of available resources, eligibility criteria should be as broad as possible. The criteria should reflect the evidence of the pattern of usage and disease burden, and the study population should reflect all well-known relevant disease characteristics that may interact with the treatment.

b) Patients with co-morbidities should not be explicitly excluded from the study enrollment, with the exception of relevant safety aspects. Both acupuncture naïve and non-naïve patients should be enrolled as well.

c) The study disease/condition should be defined as clearly as possible.

4) Patient recruitment

a) The type of comparator group chosen should take into account its possible effect on recruitment success.

b) As far as possible, recruitment strategies should be similar for all treatment arms and recruitment should be carried out in places where the respective treatments are usually employed (e.g., both CAM and conventional clinics and practitioners).

c) Sampling of study participants should be as systematic as possible, for example, by using registries or health insurance records and inviting a random sample or systematic sample of patients seeking treatment (e.g. every 50^th^ patient) from the health insurance record list.

d) Patients’ treatment preferences should be determined and efforts should be made to recruit both those who express strong treatment preferences and those who do not.

### Treatment protocol, expertise and setting

5) Acupuncture intervention

a) The acupuncture treatment protocol should to some extent reflect real-world clinical care. If acupuncture points are predefined, the point selection should be based on broad systematic consensus (e.g., expert opinion survey, consensus meetings and/or textbook survey).

b) Different acupuncture treatment protocols (standardized, semi-standardized, fully individualized) should be compared in a design with multiple treatment arms.

c) Standardized acupuncture may take Chinese pattern diagnoses into account by:(1) including only those patients who have the most frequent pattern differentiation for the condition of interest, with treatment protocol tailored to this pattern, or (2) having different pre-established standardized treatment protocols for different Chinese pattern differentiations [49].

6) Comparison groups

a) The treatment protocol for the comparison group should be based on broad systematic consensus (e.g., expert opinion survey, consensus meetings and/or textbook survey).

b) If the terms “usual care”, “standard care” or “best practice” are used to describe comparison group treatment, a detailed description must be provided, stating how these terms are defined within the trial. In addition, relevant references should be provided.

7) Treatment documentation

a) All treatments (interventions, co-interventions, over-the-counter self-medication) carried out in all groups should be documented. A variety of documentation methods and sources may contribute details (including medical records, case report forms, etc.).

### Outcomes

8) Measures

a) If no standard outcome measures for the respective disease/condition exist, or are not suitable for CER and no pilot data is available, pilot studies should be carried out to explore the value of a broad range of outcomes that may be sensitive to the condition treated and the interventions. Qualitative and quantitative research methods could be combined to identify all relevant associated outcomes.

b) The main outcome measures should focus on patient-centered outcomes and, if appropriate, include relevant biological measures for the respective disease/condition. Diagnosis-specific validated standards for outcome measures published by professional associations (e.g., the International Headache Society on headache measures [50]) or expert consensus (e.g., on back pain measures [10]) should be followed, when validated for the context of use (e.g. cultural group), and whenever possible, to permit better comparison of study results.

c) Multiple primary outcomes addressing distinct dimensions may be used, if appropriate. This should be addressed in the sample size calculation and statistical analysis plan.

d) There is great value in reporting the percentage of patients with clinically meaningful response for the main outcome measures, because this information is easily interpreted by non-scientists.

e) Secondary outcome measures should capture relevant patient-centered dimensions of the respective disease/condition (both self-reported and biological) and might, if appropriate, include measures of collateral effects (i.e., positive and negative consequences of the treatment experience, often seemingly unrelated to the main outcomes).

9) Timing

a) In chronic diseases long-term observations (at least 1 year) are highly recommended.

b) The use of periodic intervals to document and compare the trajectory and persistence of treatment effects is recommended. However, the frequency of assessment should be balanced, so that relevant information is gained without major disruptions of treatment implementations or practice setting.

### Study design and statistical analysis

10) Registries

a) The clinical trajectory of individual patients, which can be tracked by registries, could inform future acupuncture studies (e.g., as to intervention details, outcomes, safety, or characteristics of a usual care population that receives acupuncture treatment outside of a study situation).

b) When collecting registry data, information should be obtained from all types of providers who offer the services of interest.

c) Registries may be useful in identifying frequency of important potential covariates, including spirituality and beliefs, prior CAIM use, nutrition, exercise, etc., but this information is often not included.

d) Registries could serve as a recruitment platform for comparative studies.

e) If experience with registries in acupuncture research is lacking, the development and use of pilot registries may prove helpful.

11) Allocation methods

a) Use of appropriate allocation methods is strongly recommended. Randomization at the level of individual patients is still the method most frequently used, but dynamic allocation procedures (e.g. rank minimization [35]) may be used as an alternative. The final choice depends on the design of the study as a whole.

b) Stratified randomization or adaptive allocation techniques may be used to prevent imbalances for relevant covariates and potential confounders in study arms.

c) Partially randomized patient preference designs have an advantage in that they provide additional exploratory information, whether the results observed for randomized patients are different from those not randomized because of strong treatment preferences. These designs, while adding potentially important outcomes data to a clinical trial, are often not feasible because of the need for much larger sample sizes.

d) Cluster randomization is recommended by the Institute of Medicine especially for acupuncture studies and may be used if feasible [2]. When planning such a trial, it is necessary to consult the relevant literature and local institutional roles to determine from whom, when, and how informed consent must be obtained [51], and to take into account that a larger sample might be needed than in patient level randomized trials [44].

e) Standard procedures ensuring allocation concealment (e.g., central randomization or secure databases) should be employed. Recommendations for stratification will be given under point 15.

12) Blinding

a) Blinded outcome measurement (e.g., a blinded rater) is recommended in order to reduce bias, especially for outcomes that are assessed by the practitioner (e.g. range of motion as a physical assessment). Methods to minimize the risk of unblinding (e.g., allocation concealment, rater training, standardized assessment protocol) should be employed.

b) Patient-reported trial outcomes data (e.g., migraine days, quality of life) should be kept inaccessible to the practitioner (e.g., by using sealed envelopes or preferably by sending questionnaires directly to a study office independent of the intervention site or using a blinded interviewer). This should not influence the symptoms usually reported by the patients to the practitioner for treatment follow up.

13) Patient preferences and expectation

a) Patient preferences should, if appropriate, be acknowledged in the study design, e.g., by using a partially randomized patient preference design. If such a design is not feasible, then it is important to document both the patients’ preferences regarding the treatment options available in the trial as well as the degree of their knowledge about these treatment options.

b) Patient and practitioner preferences and expectations should be assessed before the intervention begins (in randomized trials before randomization).

14) Sample size

a) Sample size depends mainly on the outcome(s) of main interest and the minimum clinically important difference (MCID) for the respective outcome(s).

b) Sample size should account for greater heterogeneity in CER study populations; because of this researchers should specifically avoid conducting small trials (< 50 patients per arm) in CER, unless there is a specific reason to do such studies (e.g., pilot studies to test feasibility and recruitment).

15) Subgroups

a) Relevant subgroups for the disease/condition under study should be identified based on existing data and the literature. Subgroup analysis should be done at least for gender, because there is preliminary evidence that acupuncture might be more effective in women than men [52]. Also of interest are subgroup analyses for different Chinese pattern diagnoses and for acupuncture patients, who are naïve/non-naive to acupuncture. Further analyses could be carried out for age, ethnicity, disease severity/duration, treatment preference and recruitment site.

b) The main subgroup analyses should be pre-specified in the analysis plan and included in sample size planning for confirmatory testing. Further subgroup analyses could be done on an exploratory level, but should be stated as an objective in the study protocol.

16) Statistical analysis

a) Primary analysis for trials on superiority of acupuncture should be intention-to-treat. In order to assess real-world effectiveness of treatments, benefits and harms should be judged and compared according to the treatment to which patients were assigned.

b) Analyses should adjust for relevant potential confounders (e.g., baseline value of the outcome measure, stratification variables, expectation).

c) Especially in non-randomized studies (e.g. observational data from registries), procedures to compensate for baseline differences must be used (e.g., matching and/or adjusted analysis).

### Economic evaluations

17) Relevance

a) Comparing the effectiveness of treatment options should be the primary aim of CER, but economic evaluations should be included whenever possible as a secondary aim.

b) To allow realistic cost estimates, the setting(s) of the study should reflect as closely as possible the real-world clinical practice setting for each intervention. If a study includes a standardized and a non-standardized acupuncture arm, it would be useful to compare their cost-effectiveness.

18) Methodological approach

a) Standard methods for economic evaluations should be employed, and effectiveness measures that include both benefits and harms (e.g., utility measures based on SF-36, SF-12 or EQ-5D) should be used [48].

b) Sensitivity analysis should include, whenever possible, different stakeholder perspectives (e.g., society, payer and patient). Because acupuncture is often paid out-of-pocket, the patient’s perspective is highly relevant.

c) Subgroup analysis should mainly follow the subgroups defined a priori for the effectiveness study. A subgroup analysis for gender is recommended, because there is preliminary evidence that gender may influence the cost-effectiveness of acupuncture treatment [53].

d) Exploratory analyses of factors that predict a better treatment response are suggested to develop future hypotheses.

19) Observation time

a) In chronic diseases long-term observations (at least 1 year) with intermediate measurement time points are highly recommended, in order to evaluate cost-effectiveness as developed over time.

### Publication

20) Existing guidelines

To ensure that CER on acupuncture will fulfill reporting standards, the relevant CONSORT guidelines should be consulted and followed:

CONSORT for parallel group randomized trials [13].

CONSORT extension for cluster randomized trials [15].

CONSORT extension for pragmatic trials [8].

CONSORT for non-pharmacological trials [14].

STRICTA as CONSORT extension for acupuncture studies [16].

21) Content

a) Publication of a detailed study protocol (design publication) should take place whenever possible prior to publication of study results.

b) The study should be registered in an international accessible trial database with as many details as possible provided.

c) Publication of the completed study should describe why and how it qualifies as CER and make clear the phase of the study.

d) The setting of the study should be described, including information about the typical usual care setting in the country where the study was performed (and if relevant in other countries). The procedure for selection of practitioners for each treatment group should be described, with an account of whether and how those included in the study differ from the average practitioner (e.g. training, experience).

e) Wording of treatment group descriptions in the informed consent should be provided.

f) If a usual-care or standard-care comparison group is used, a detailed description with citations should be included in the intervention section.

g) Detailed results of all treatments, adherence, and co-interventions in the different groups should be provided.

h) The most relevant subgroup analyses and analyses of patient characteristics that predict a better outcome should be published together with primary results. Detailed subgroup analysis and/or de-identified patient level data could be provided as online files.

## Discussion

This is the first EGD in the field of Complementary, Alternative and Integrative Medicine and it offers many potential benefits. During the development process, a broader understanding of the unique methodological aspects of CER emerged in the stakeholder group. CER studies are intended to improve the external validity of clinical research to enable decision makers to make informed decisions. Also, EGDs can contribute to a more strategic use of limited research resources and more consistency in trial design.

While other documents provide guidance for publishing studies (e.g. CONSORT statement) EGDs provide recommendations for the design of future studies.

This acupuncture EGD derived from a systematic development process, and the active involvement of different stakeholders who have experience with acupuncture (clinicians, patients, payers and researchers). Furthermore, an additional international expert review with eight acupuncture research experts from four countries enhanced the content and quality of the present EGD.

That stakeholders living in Asian countries were not involved in the development of this EGD might be seen as a limitation, but to date, CER has had its main focus in the United States, and is mainly known in Western countries. In addition, a consensus procedure using a web-based survey method might have allowed even broader and more heterogeneous contributions from stakeholders in Eastern as well as Western countries. That this EGD focuses on acupuncture and covers CER for acupuncture independent of condition treated has the shortcoming that the recommendations on outcomes are not at the disease level. However, the aim of this EGD is to provide a single comprehensive guideline for future acupuncture research on the seven fundamental methodological areas: (1) CER study design, (2) treatment protocol, (3) expertise and setting, (4) outcomes, (5) study design and statistical analyses, (6) economic evaluation, and (7) publication. The feasibility of the EGD to design studies was tested in February and April 2012 in two CER research methodology courses with 20–25 participants each, one with US master students and the other with an international audience of researchers.

## Conclusion

For the first time, the present EGD provides systematic methodological guidance for future CER on acupuncture. This is the first EGD in the field of Integrative medicine and further EGDs are planned and many aspects of this EGD might be transferrable to other non-pharmacological interventions in the field of complementary and integrative medicine.

## Competing interests

The work shop was funded by The Institute for Integrative Health a non for profit organization, Brian Berman is the president of the Institute for Integrative Health and Claudia Witt received a travel grant for the submitted work; there was no financial relationships with any organizations that might have an interest in the submitted work in the previous 3 years; there are no other relationships or activities that could appear to have influenced the submitted work.

## Authors’ contributions

Wrote the first draft of the paper: CMW. Contributed to the results, the writing of the paper and the Delphi rounds and agreed to the final version: all authors. Reviewed the paper and gave useful comments and agreed on the final version: all collaborators. CMW and BB are guarantors for the paper and accept full responsibility for the work and controlled the decision to publish.

## Pre-publication history

The pre-publication history for this paper can be accessed here:

http://www.biomedcentral.com/1472-6882/12/148/prepub
